# Supplementary material for: The m7G Reader NCBP2 Promotes Pancreatic Cancer Progression by Upregulating MAPK/ERK Signaling
Source: Cancers (Basel). 2023 Nov 17;15(22):5454. doi: 10.3390/cancers15225454 (PMC10670634; doi:10.3390/cancers15225454)
Supplement: Supplementary file 1 [file cancers-15-05454-s001.zip › Supplementary Figures.pdf]

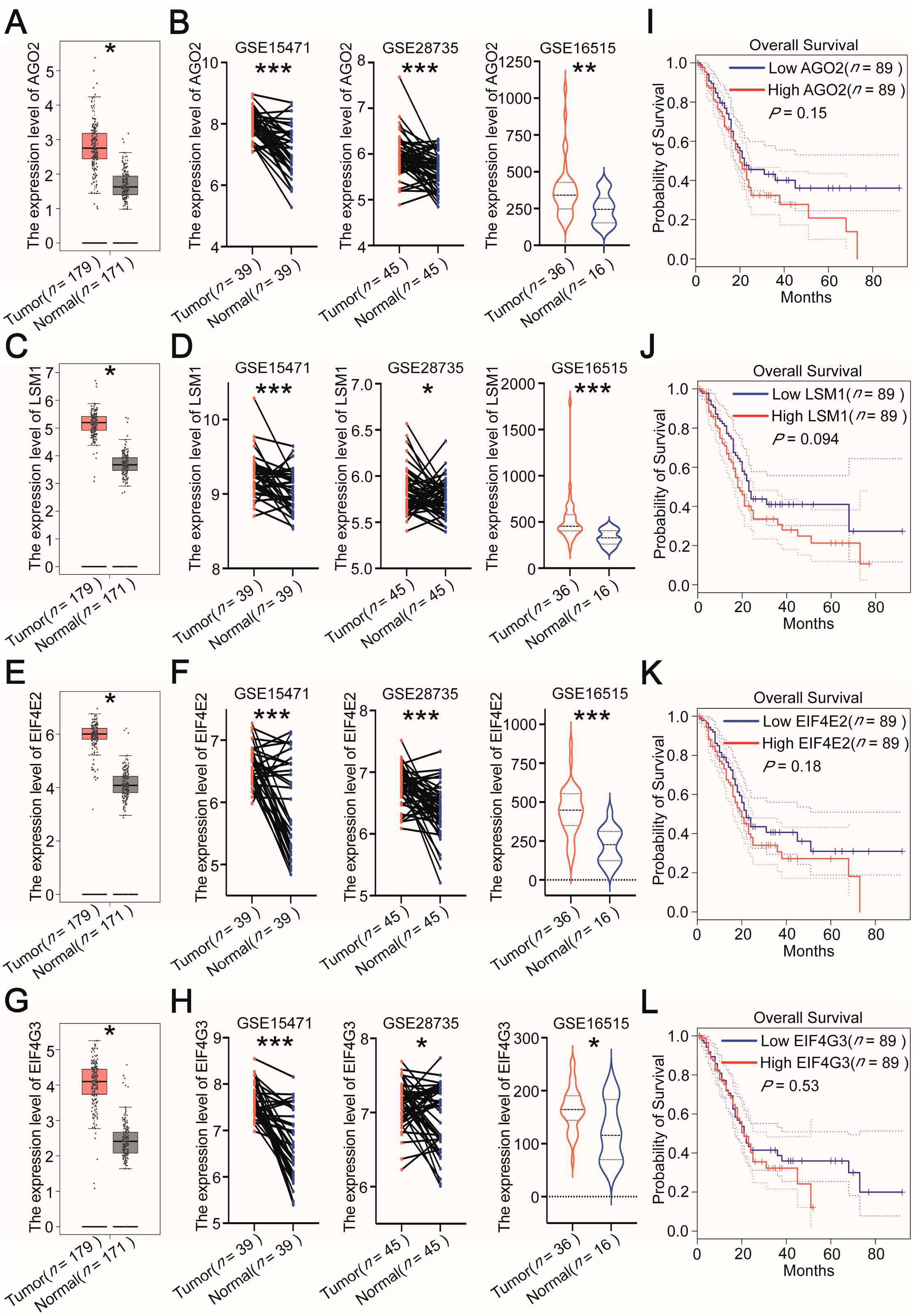

Figure S1. The expression levels and associated survival analysis of m[7]G regulator AGO2, LSM1, EIF4E2 and EIF4G3 in PDAC patients from GEPIA 2.0 and GEO cohorts. (**A**) Analysis of AGO2 transcript levels in PDAC and normal tissues based on the RNA-seq data from GEPIA 2.0-PDAC cohort. (**B**) Analysis of AGO2 transcript levels in PDAC and normal tissues of PDAC patients from GEO cohorts (GSE15471, GSE28735, GSE16515). (**C**) Analysis of LSM1 transcript levels in PDAC and normal tissues based on the

RNA-seq data from GEPIA 2.0-PDAC cohort. (**D**) Analysis of LSM1 transcript levels in PDAC and normal tissues of PDAC patients from GEO cohorts (GSE15471, GSE28735, GSE16515). (**E**) Analysis of EIF4E2 transcript levels in PDAC and normal tissues based on the RNA-seq data from GEPIA 2.0-PDAC cohort. (**F**) Analysis of EIF4E2 transcript levels in PDAC and normal tissues of PDAC patients from GEO cohorts (GSE15471, GSE28735, GSE16515). (**G**) Analysis of EIF4G3 transcript levels in PDAC and normal tissues based on the RNA-seq data from GEPIA 2.0-PDAC cohort. (**H**) Analysis of EIF4G3 transcript levels in PDAC and normal tissues of PDAC patients from GEO cohorts (GSE15471, GSE28735, GSE16515). (**I**) Overall survival for PDAC patients with high or low AGO2 expression in GEPIA 2.0-PDAC cohort. (**J**) Overall survival for PDAC patients with high or low LSM1 expression in GEPIA 2.0-PDAC cohort. (**K**) Overall survival for PDAC patients with high or low EIF4E2 expression in GEPIA 2.0-PDAC cohort. (**L**) Overall survival for PDAC patients with high or low EIF4G3 expression in GEPIA 2.0-PDAC cohort, $*P < 0.05$, $**P < 0.01$, and $***P < 0.001$.

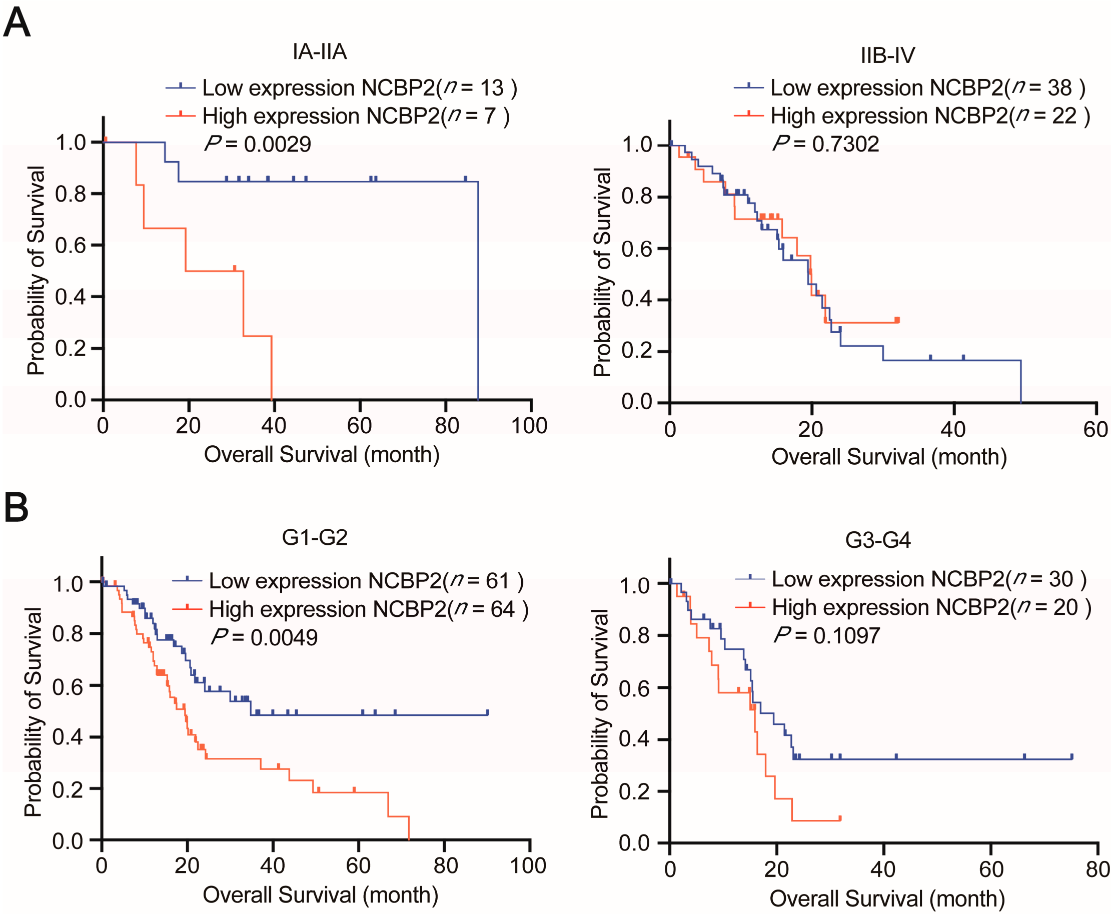

Figure S2. NCBP2 is correlated with poor prognosis in the early stages of PDAC. (**A**) Overall survival curves for IA-IIA and IIB-IV stage PDAC patients with high or low NCBP2 expression in TCGA cohort. (**B**) Overall survival curves for G1-G2 and G3-G4 PDAC patients with high or low NCBP2 expression in TCGA cohort.

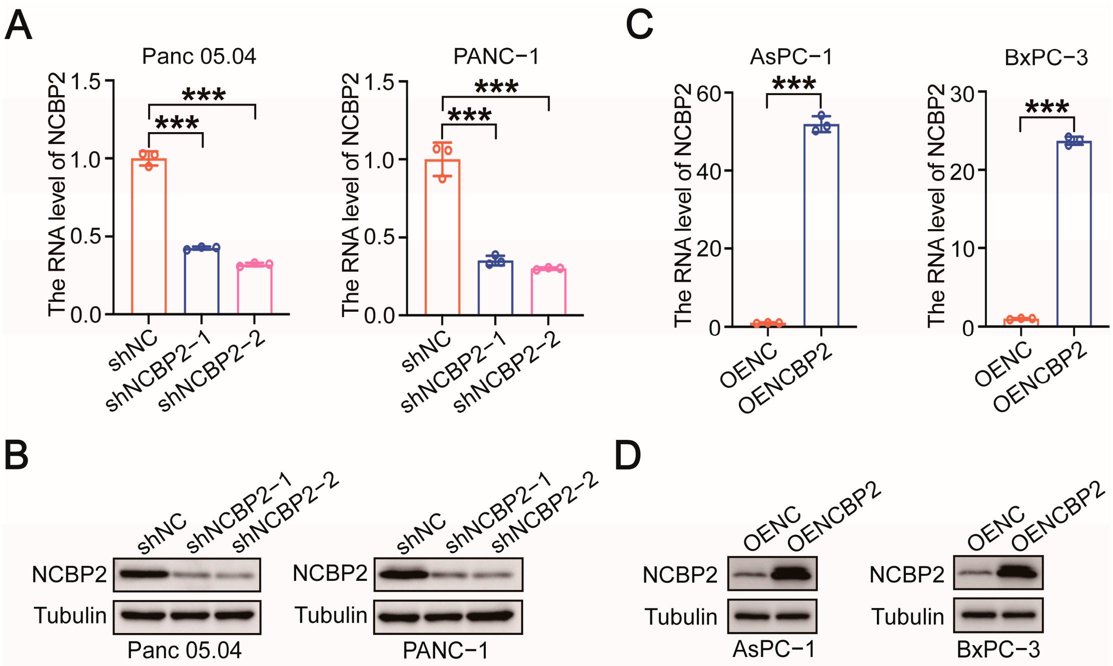

Figure S3. Establishing stable NCBP2-knockdown and NCBP2-overexpression PDAC cell lines. (**A-B**) The RNA and protein expression level of NCBP2 in control and NCBP2-knockdown Panc 05.04 and PANC-1 cells. (**C-D**) The RNA and protein expression level of NCBP2 in control and NCBP2-overexpression AsPC-1 and BxPC-3 cells, ***P < 0.001.

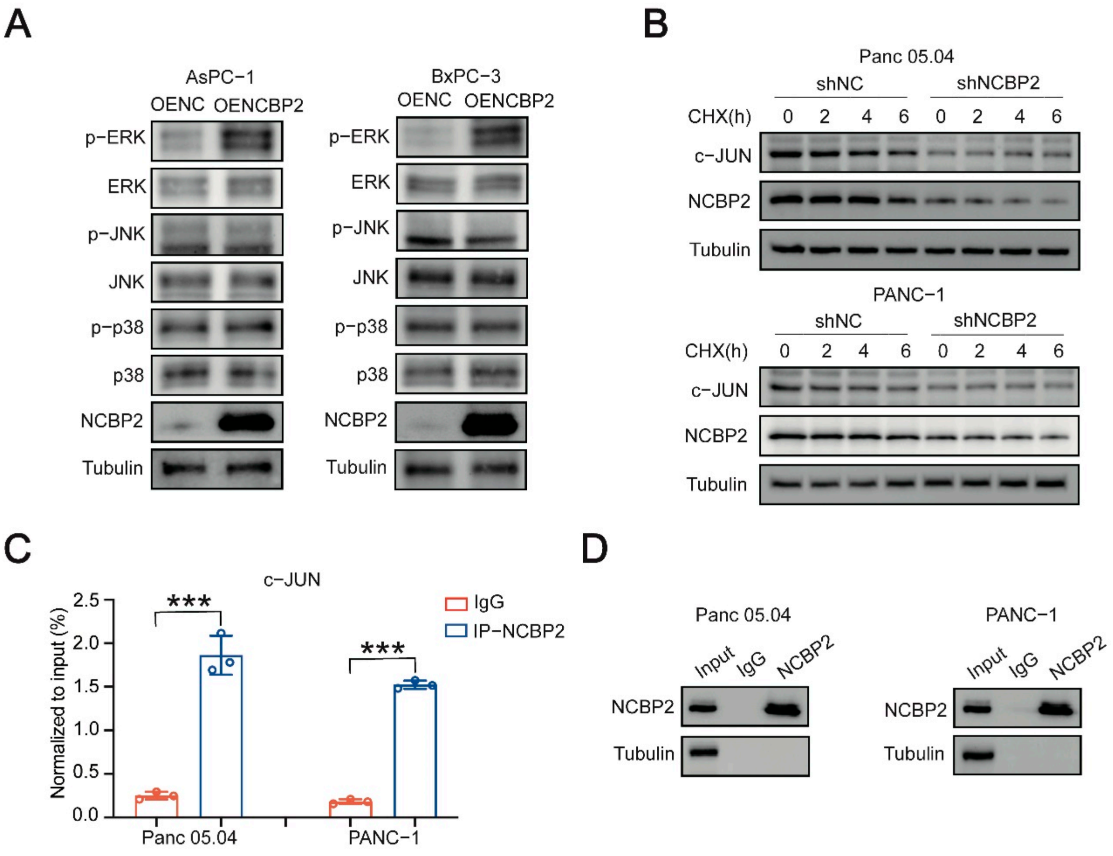

Figure S4. NCBP2 up-regulates c-JUN and activates MEK/ERK signaling in a m7G- dependent manner. (**A**) Immunoblotting for protein levels of total JNK/phosphorylated JNK (Thr183/Tyr185), total

p38/phosphorylated p38 (Thr180/Tyr182), and total ERK/phosphorylated ERK (Thr202/Tyr204) in control and NCBP2-overexpression PDAC cells. Tubulin was used as the internal control. (**B**) Immunoblotting analysis of NCBP2 and c-JUN expression. Tubulin was used as the internal control. (**C**) The mRNA expression levels of c-JUN from IgG and NCBP2 RIP samples in Panc 05.04 and PANC-1 cells. (**D**) Immunoblotting for protein levels of NCBP2 from IgG and NCBP2 RIP samples in Panc 05.04 and PANC-1 cells. Tubulin was used as the control, ***$P < 0.001$.

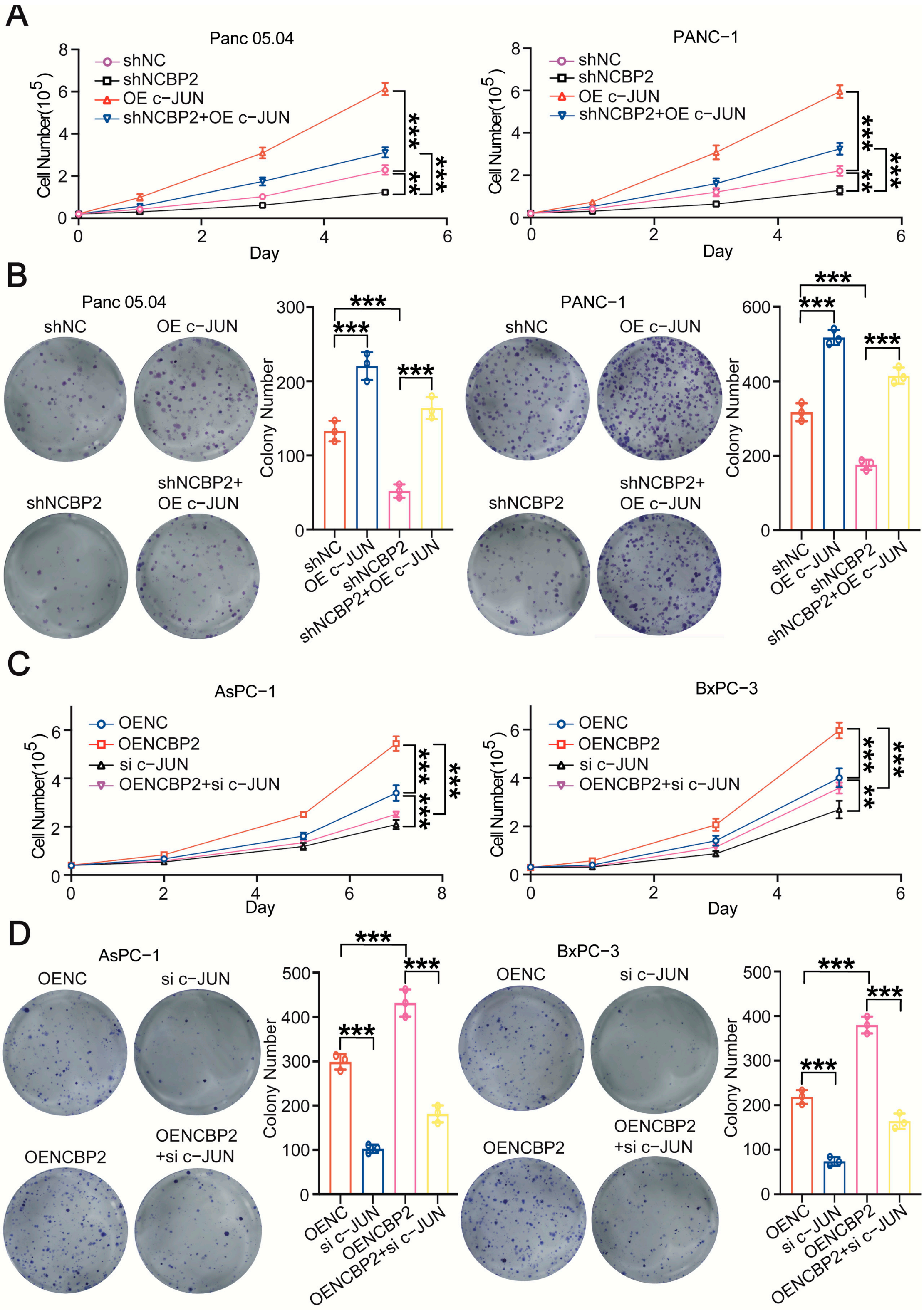

Figure S5. NCBP2 promotes PDAC progression by activating c-JUN/MEK/ERK signaling pathway. (**A-B**) The cell counting and colony formation assay were performed after overexpressing c-JUN in control and NCBP2-knockdown PDAC cells. (**C-D**) The cell counting and colony formation assay were performed after c-JUN-knockdown in control and NCBP2-overexpressed PDAC cells, $**P < 0.01$, and $***P < 0.001$.
