# Supplementary material for: The m7G Reader NCBP2 Promotes Pancreatic Cancer Progression by Upregulating MAPK/ERK Signaling
Source: Cancers (Basel). 2023 Nov 17;15(22):5454. doi: 10.3390/cancers15225454 (PMC10670634; doi:10.3390/cancers15225454)
Supplement: Supplementary file 1 [file cancers-15-05454-s001.zip › Supplementary Tables.pdf]

Table S1. Online analysis website.

website	URL
GEPIA 2.0	http://gepia2.cancer-pku.cn/#general
TCGA-PDAC	http://www.cbioportal.org/study/clinicalData?id=paad_tcga_pan_can_atlas_2018
GSE15471	https://www.ncbi.nlm.nih.gov/geo/
GSE16515	https://www.ncbi.nlm.nih.gov/geo/
GSE28735	https://www.ncbi.nlm.nih.gov/geo/

Table S2. Primer sequences for qPCR, siRNAs sequences, primers for NCBP2-targeting shRNA, NCBP2-overexpression and c-JUN-overexpression plasmids construction used in this study.

**Primers used for quantitative qRT-PCR**		
**Name**	**Forward-primer**	**Reverse-primer**
NCBP2	AAAACGCCATGCGGTACATAA	GCCTGCCCTCCTTAAAGCC
c-JUN	TGGGTTACACCGAAGATGCC	AATTGGATACCAGCGGAGGC
18s	CTACCACATCCAAGGAAGCA	TTTTTCGTCACTACCTCCCCG
**Primers used for plasmid construction**		
**Name**	**Forward-primer**	**Reverse-primer**
NCBP2-CDS	CCCGGACGAATTCTTCGAA ACCATGATGTCGGGTGGCCTCCTG	TGCGGATCACTAGTGCTAGCTCACTGGTTCTGTGCCAGTTTTC
c-JUN-CDS	CCCGGACGAATTCTTCGAAACCATGATGACTGCAAAGATGGAAACGAC	TGCGGATCACTAGTGCTAGCTCAAAATGTTTGCAACTGCTGC
**Primers used for shRNA construction**		
**Name**	**Forward-primer**	**Reverse-primer**
shNCBP2-1	CCGG—ATCATTATGGGTCTGGATAAA—CTCGAG—TTTATCCAGACCCATAATGAT—TTTTTG	AATTCAAAAA—ATCATTATGGGTCTGGATAAA—CTCGAG—TTTATCCAGACCCATAATGAT
shNCBP2-2	CCGG—GTGACAATGAAGAACAAGAAA—CTCGAG—TTTCTTGTTCTTCATTGTCAC—TTTTTG	AATTCAAAAA—GTGACAATGAAGAACAAGAAA—CTCGAG—TTTCTTGTTCTTCATTGTCAC
**Sequence for siRNA**		
c-JUN-siRNA-1	TAGTACTCCTTAAGAACACAA	
c-JUN-siRNA-2	CGGACCTTATGGCTACAGTAA	

Table S3. Primary antibodies used in this study.

Name	Company	Catalog Number	Assay
NCBP2	ThermoFisher	#PA5-75234	WB (1:1000), IHC (1:100), IP (1:50)
c-JUN	Cell Signaling Technology	#9165S	WB (1:1000), IHC (1:300)
ERK	Cell Signaling Technology	#9926T	WB (1:1000)
JNK	Cell Signaling Technology	#9926T	WB (1:1000)
P38	Cell Signaling Technology	#9926T	WB (1:1000)
p-ERK	Cell Signaling Technology	#9910T	WB (1:2000), IHC (1:200)
p-JNK	Cell Signaling Technology	#9910T	WB (1:1000)
p-p38	Cell Signaling Technology	#9910T	WB (1:1000)
MEK	Proteintech Group	#11049-1-AP	WB (1:1000)
p-MEK	Cell Signaling Technology	#2338S	WB (1:2000)
Tubulin	Proteintech Group	#66031-1-Ig	WB (1:5000)
7-Methylguanosine	Biovision	#BIV-6655-100	Me-RIP (1:1000)
Ki67	Servicebio	#GB111499	IHC (1:400)
Anti-rabbit IgG, HRP-linked Antibody 7074	Cell Signaling Technology	#7074S	IP (1:5000)
HRP, Goat Anti-mouse IgG	Abbkine	#A21010	IP (1:400)
